# UVA and UVB Irradiation Differentially Regulate microRNA Expression in Human Primary Keratinocytes

**DOI:** 10.1371/journal.pone.0083392

**Published:** 2013-12-31

**Authors:** Anne Kraemer, I-Peng Chen, Stefan Henning, Alexandra Faust, Beate Volkmer, Michael J. Atkinson, Simone Moertl, Ruediger Greinert

**Affiliations:** 1 Institute of Radiation Biology, Helmholtz Center Munich, Neuherberg, Germany; 2 Department Molecular Cell Biology, Center of Dermatology, Elbekliniken Stade/Buxtehude, Buxtehude, Germany; 3 Radiation Biology, Technical University of Munich, München, Germany; University of Tennessee, United States of America

## Abstract

MicroRNA (miRNA)-mediated regulation of the cellular transcriptome is an important epigenetic mechanism for fine-tuning regulatory pathways. These include processes related to skin cancer development, progression and metastasis. However, little is known about the role of microRNA as an intermediary in the carcinogenic processes following exposure to UV-radiation. We now show that UV irradiation of human primary keratinocytes modulates the expression of several cellular miRNAs. A common set of miRNAs was influenced by exposure to both UVA and UVB. However, each wavelength band also activated a distinct subset of miRNAs. Common sets of UVA- and UVB-regulated miRNAs harbor the regulatory elements GLYCA-nTRE, GATA-1-undefined-site-13 or Hox-2.3-undefined-site-2 in their promoters. In silico analysis indicates that the differentially expressed miRNAs responding to UV have potential functions in the cellular pathways of cell growth and proliferation. Interestingly, the expression of miR-23b, which is a differentiation marker of human keratinocytes, is remarkably up-regulated after UVA irradiation. Studying the interaction between miR-23b and its putative skin-relevant targets using a Luciferase reporter assay revealed that RRAS2 (related RAS viral oncogene homolog 2), which is strongly expressed in highly aggressive malignant skin cancer, to be a direct target of miR-23b. This study demonstrates for the first time a differential miRNA response to UVA and UVB in human primary keratinocytes. This suggests that selective regulation of signaling pathways occurs in response to different UV energies. This may shed new light on miRNA-regulated carcinogenic processes involved in UV-induced skin carcinogenesis.

## Introduction

MicroRNAs (miRNAs) comprise a family of small non-translated RNAs (∼19–24 nt) that are expressed in animals, plants and viruses [1], [2]. Their primary biological action is the adjustment of protein translation through the specific regulation of target mRNAs. The association between complementary sequence motifs in the microRNA and the 3' untranslated region (3'UTR) of target mRNAs results in the inhibition of translation [3], [4] or enhanced degradation of the target mRNAs [5], [6]. It has been estimated that more than 30% of the protein-coding transcriptome (mRNAs) is regulated by miRNAs [7], [8]. More than 2500 (miRBase, www.mirbase.org) candidate miRNAs/miRNA-precursors have been identified to date in human cells [9].

The miRNA-mediated regulation of the cellular transcriptome has been implicated as an important epigenetic mechanism for cellular pathways related to cancer development, progression and metastasis [10], [11]. Indeed, microRNAs are differentially expressed in a number of tumors [12], [13], including those affecting the skin [14].

Skin cancer, including basal cell carcinoma (BCC), cutaneous squamous cell carcinoma (SCC) and malignant melanoma (MM) is the most frequent cancer in the caucasian population. As the incidences of skin cancer are increasing more rapidly than for any other cancers [15], [16], it is important to understand the etiology of all types of skin cancer. Since UV irradiation is the main risk factor for skin cancer induction the International Agency for the Research on Cancer (IARC) has classified solar UV (UVB = 280–315 nm and UVA = 315–400 nm), as well as artificial UV radiation used in sun beds as a category 1a carcinogen (“carcinogenic to humans”) [17].

UV radiation causes mutations indicative of the misrepair of UV-induced cyclobutane pyrimidine dimers (CPD). These are the most predominant, pre-mutagenic DNA-lesions produced by both UVB and UVA irradiation of human skin cells [18], [19]. Biochemical studies on signaling pathway activation revealed that there are differently as well as similarly changed signaling pathways after UVA and UVB irradiation in keratinocytes (Syed *et al*., 2012). Phosphorylation of JNK1/2 at Thr138/Tyr185 or STAT3 at Ser727, for example, is specifically induced by UVB, while phosphorylation of AKT at Thr308 is induced only by UVA but not by UVB. On the other hand UVA and UVB both lead to increased phosphorylation of ERK1/2 at Thr202/Tyr204 or of p38 at Thr180/Tyr204 [20].

Only a limited number of investigations deal with the effect of UV-irradiation in relation to miRNA expression. Pothof *et al*. showed that miRNA-mediated gene silencing modulates the UVC-induced DNA-damage response [21]. Guo *et al*. investigated UVB-regulated miRNAs in the mouse cell line NIH3T3 [22]. Dziunycz *et al*. investigated the expression of miR-21, miR-203 and miR-205 after UVA and UVB irradiation in human keratinocytes [14]. One recent short communication listed global miRNA expression changes in human keratinocytes after UVB irradiation [23].

Changes in miRNA expression have been shown to be associated with induction and progression of malignant melanoma, the most lethal form of skin cancer [24], [25]. For the other two important types of skin cancer, the keratinocyte-derived BCC and SCC, only sparse data documenting altered miRNA expression exist [14], [26], [27]. As a consequence, the potential role of miRNAs in the etiology of skin carcinogenesis is still poorly understood. We have therefore investigated whether the expression of miRNAs in primary human keratinocytes is influenced by UVA and UVB exposure. We have identified a general miRNA response to UV, coexisting with wavelength-specific miRNA responses. The identification of potential regulatory targets that are involved in skin cancer development and/or progression indicates that the miRNA regulation plays a significant role in skin carcinogenesis.

## Results and Discussion

### miRNA expression changes induced by UVA and UVB radiation

**Figure 1:**
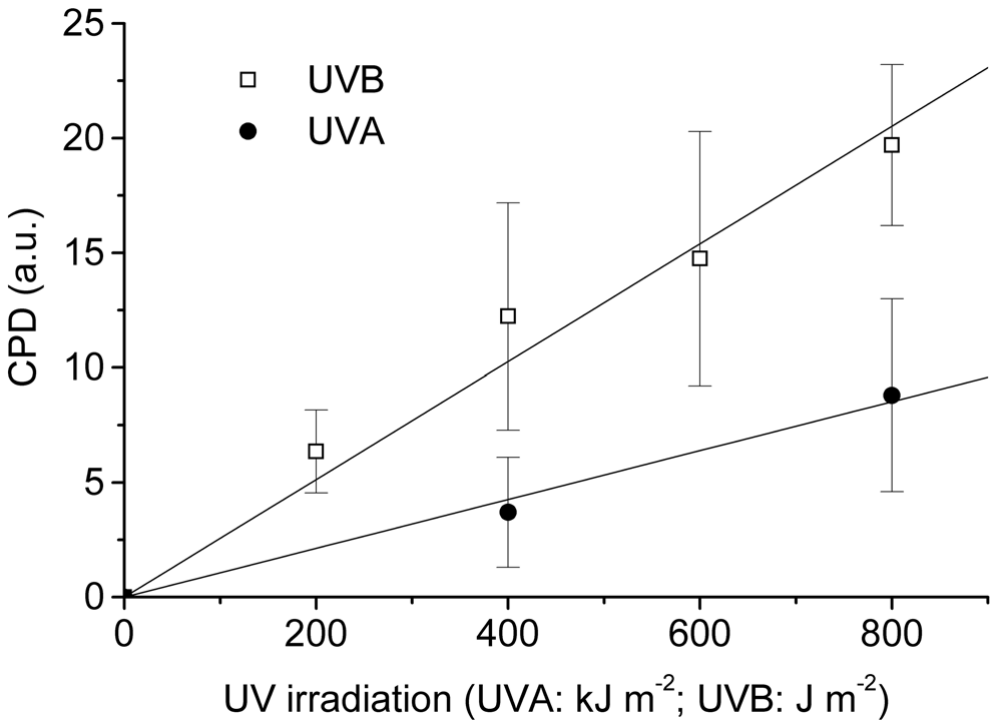
Dose dependent flow cytometric detection of cyclobutane pyrimidine dimer (CPD) production after UVA or UVB irradiation in human primary keratinocytes. Irradiated cells were fixed immediately after irradiation and then labeled with monoclonal antibodies against CPDs. Cellular CPD immuno-fluorescence (FITC) was measured in arbitrary units [a.u.].

In our investigation we focused on a comparison of the effect of UVA and UVB irradiation on miRNA expression 6h after UV doses (600 kJ/m^2^ UVA; 300 J/m^2^ UVB) that produce comparable levels of DNA damage in the form of cyclobutane pyrimidine dimers (6.3 arbitrary unit (a.u.) after 600 kJ/m^2^ UVA versus 7.5 a.u. after 300 J/m^2^ UVB; Fig 1). These exposures are comparable to those that can be received by human skin on a typical sunny day [28].

**Figure 2:**
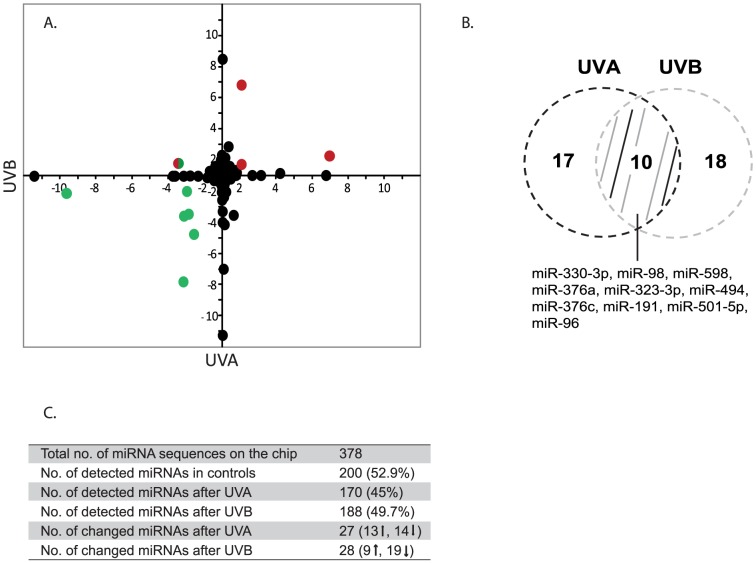
Comparison of UVA and UVB deregulated miRNAs 6h after irradiation. (A) miRNAs up-regulated (red) or down-regulated (green) after UVA and UVB are colored. (B) Venn diagram shows the overlap between UVA- and UVB-regulated miRNAs. (C) Summary of miRNA analysis of human primary keratinocytes from two female donors after UVA and UVB irradiation. Deregulated miRNAs display a p-value < 0.05 (one sample t-test).

The TaqMan Low Density Array (TLDA) used in this investigation is aimed to detect 378 mature human miRNAs. Two hundred miRNAs (52.9%) could be detected in control (mock irradiated) human primary keratinocytes (Fig 2C).

In UVA-irradiated cells 170 miRNAs were detected on TLDA. Of these 27 were differentially expressed compared to non-irradiated cells (13 up- and 14 down-regulated) (Fig 2B). The increase in expression ranged from +1.7 to +7.0 and down-regulation between –1.8 and –11.6. The most highly up-regulated miRNAs were miR-23b (+6.8) and miR-376c (+7.0), while miR-494 (–9.8) and miR-487b (–11.6) were the most down-regulated miRNAs (Table 1).

**Table 1:** miRNA expression changes in primary human keratinocytes 6h after UVA irradiation (^†^also changed after UVB).

microRNA	n-fold change	p-value	chromosomal localization
*up-regulated*			
miR-23b	6.8	2.50E-06	9q22.32
miR-96^†^	1.7	0.002	7q32.2
miR-132	2.7	0.002	17p13.3
miR-191^†^	2.1	0.015	3p21.31
miR-196b	1.8	0.017	7p15.2
miR-224	1.8	0.043	Xq28
miR-340	3.2	0.016	5q35.3
miR-376c^†^	7.0	0.011	14q32.31
miR-452	4.2	0.009	X q28
miR-484	1.7	0.008	16p13.11
miR-501-5p^†^	2.1	0.035	Xp21.23
miR-574-3p	1.7	0.002	4p14
miR-886-5p (VTRNA2-1)*	1.7	0.040	5q31.2
*down-regulated*			
miR-10a	–3.8	3.80E-06	17q21.32
miR-18b	–3.0	1.80E-06	Xq26.2
miR-98^†^	–3.1	1.40E-10	Xp21.22
miR-99b	–2.1	4.00E-04	19q13.41
miR-127-3p	–1.8	3.70E-06	14q32.2
miR-130b	–2.6	2.50E-09	22q11.21
miR-210	–1.9	5.00E-04	11p15.5
miR-212	–1.8	2.30E-06	17p13.3
miR-323-3p^†^	–3.4	7.40E-08	14q32.31
miR-330-3p^†^	–2.8	2.30E-08	19q13.32
miR-376a^†^	–3.4	2.00E-04	14q32.31
miR-487b	–11.6	9.10E-13	14q32.31
miR-494^†^	–9.8	7.50E-17	14q32.31
miR-598^†^	–3.2	4.60E-07	8p23.21

: miR-886-5p is a fragment of vault RNA (VTRNA2-1) [60].

In UVB-irradiated cells 188 miRNAs were evaluable on TLDA. After UVB irradiation 28 deregulated miRNAs were identified (9 up- and 19 down-regulated) (Fig. 2B) with expression changes ranging from +1.7 to +8.4 for increases and ranges from –1.9 to –25.0 for decreases. The highest up-regulation was detected for miR-501-5p (+6.8) and for miR-361-5p (+8.4). miR-23a (–25.0) and miR-323-3p (–7.7) showed the strongest reductions (Table 2).

**Table 2:** miRNA expression changes in primary human keratinocytes 6h after UVB irradiation (^†^also changed after UVA).

microRNA	n-fold change	p-value	chromosomal localization
*up-regulated*			
let-7c	2.3	0.016	21q21.1
miR-139-5p	2.1	0.0027	11q13.4
miR-191^†^	1.7	5.70E-08	3p21.31
miR-339-3p	2.0	0.003	7p23.3
miR-361-5p	8.4	0.0058	Xq21.2
miR-362-5p	2.8	0.019	Xp11.23
miR-376c^†^	2.3	0.0017	14q32.31
miR-455-3p	2.0	0.013	9q32
miR-501-5p^+^	6.8	0.006	Xp11.23
*down-regulated*			
miR-20b	–2.0	1.40E-05	Xq26.2
miR-23a	–25.0	0.006	19p13.13
miR-29c	–2.2	1.99E-06	1q32.3
miR-30c	–2.0	5.80E-05	1p34.2
miR-96^†^	–3.5	5.90E-17	7q32.2
miR-98^†^	–3.4	5.40E-06	Xp11.22
miR-181c	–7.0	1.00E-13	19p13.13
miR-218	–2.0	8.50E-11	4p15.31
miR-301a	–1.9	2.00E-04	17q22
miR-323-3p^†^	–7.7	4.40E-13	14q32.31
miR-330-3p^†^	–4.7	1.00E-10	19q13.32
miR-335	–6.9	5.31E-15	7q32.2
miR-376a^†^	–3.5	1.70E-08	14q32.31
miR-411	–3.3	1.30E-07	14q32.31
miR-494^†^	–2.0	1.90E-12	14q32.31
miR-503	–4.0	1.50E-15	Xq26.3
miR-532-5p	–4.1	4.80E-19	Xp11.23
miR-598^†^	–1.9	5.80E-10	8p23.1
miR-660	–2.4	1.60E-07	Xp11.23

Of the UV-regulated miRNAs 10 were found to be regulated by both UVA and UVB. This means that about 30% of the miRNAs affected by UV radiation were modulated by both radiation qualities. It has to be noted that, except for miR-96 (+1.7, UVA; –3.5, UVB) (Fig 2A), expression changes of these miRNAs are in the same direction for both radiation qualities. Thus miR-98, miR-323-3p, miR-330-3p, miR-376a, miR-494, miR-598 were down-regulated by UVA and UVB, while miR-191, miR-376c and miR-501-5p were up-regulated by both. The highest up-regulated miRNAs are miR-376c (+7.0, UVA; +2.3, UVB) and miR- 501-5p (+2.1, UVA; +6.8, UVB). Down-regulation was most pronounced in miR-494 (–9.8, UVA; –2.0, UVB) and miR-323-3p (–3.4, UVA; –7.7, UVB) (see Table 1, 2 and Fig. 2). Interestingly, of these ten commonly regulated microRNAs miR-98, miR-191, miR-323-3p, miR-330-3p, miR-494, and miR-598 were reported to be also deregulated after ionizing radiation [29], [30], [31] and miR-376a was shown to be a regulator of apoptosis in response to arsenic trioxide treatment [32]. The regulation by further DNA damaging stressors might suggest a common involvement of this set of microRNAs in DNA damage response.

Some of the UV-regulated miRNAs identified in our study were shown to be UV-triggered in previous reports. For example, an up-regulation of let-7c after UVB exposure was also found by Zhou *et al*. [23]. An up-regulation of miR-23b and a down-regulation of miR-98 were detected by Pothof *et al*. after UVC [21]. The regulation of miR-23a, miR-96 and miR-98 in our study is opposite to the regulation reported by Zhou *et al.* (2012). Whether this discrepancy is due to different time points (4h versus 6h in the present study), different irradiation sources, inter-individual differences in keratinocytes or other causes, is unknown.

The identification of miRNAs individually or commonly regulated in response to UVA and UVB suggests different and joint response pathways triggered by miRNAs. This is in line e.g. with the work of Syed *et al*. who showed [20] differently and similarly changed signaling pathways after UVA and UVB irradiation in keratinocytes.

### qPCR confirmation of differentially expressed miRNAs after UV-irradiation

**Figure 3:**
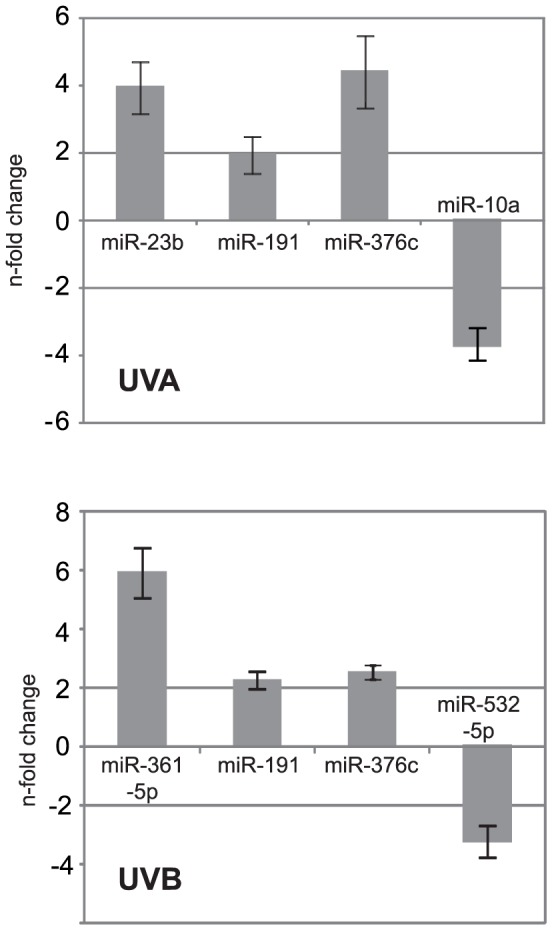
Single primer RT-PCR validation of UVA- and UVB-regulated miRNAs. The deregulation of selected UV-responsive miRNAs (identified by TaqMan Low Density Arrays) was done by using miRNA specific primer setups followed by the normalization to RNU44.

miRNA expression changes, detected on the microarray (TLDA), were validated by qPCR for a couple of miRNAs by using specific primers for cDNA synthesis and real-time PCR (Fig. 3). In detail we confirmed the up-regulation of miR-23b (UVA), miR-361-5p (UVB), miR-191 (UVA and UVB) and miR-376c (UVA and UVB). Furthermore, the down-regulation of miR-10a (UVA) and miR-532-5p (UVB) was confirmed.

### Genomic context of genes encoding differentially expressed miRNAs

Chromosomal mapping of genes encoding for differentially expressed miRNAs showed the involvement of loci on 15 different chromosomes in the UV irradiation response (see Table 1 and 2). About 20% of miRNAs that were differentially expressed after UVA or UVB irradiation map to the 14q32.31 miRNA cluster. This locus is regarded as the largest human tumor suppressor miRNA cluster [33]. About 20% and 29% of UVA- or UVB-regulated miRNAs, respectively, were found on the X chromosome. miRNAs located on chromosome X have been reported to be associated with cancer development or progression [34]. Whether the clustering of UV-regulated miRNAs on certain chromosomes is just reflecting the limited number of miRNAs studied (378 out of about presently known 2500 human miRNAs) or whether there is involvement of specific miRNA clusters in the UV response should be elucidated in further investigations.

### Identification of common regulatory elements in promoter of UV-deregulated miRNAs

In order to identify possible regulatory factors that modulate miRNA expression after UV-irradiation we looked for regulatory elements in the promoter regions of differentially expressed miRNAs using the NSITE program (see Materials & Methods).

Common sets of transcription factor binding sites were found in the promoter of subsets of miRNAs sharing a common response to both UVA and UVB. Of the 3 up-regulated miRNAs after UVA- and UVB-irradiation (miR-191, mir-376c, miR-501) miR-191 and miR-376c shared the same regulatory element GLYCA-nTRE (gcaggtgaggacttca), which belongs to thyroid regulatory elements (TREs*),* whereas miR-376c and miR-501-5p shared GATA-1-undefined-site-13 (cccacccac). Among the 6 down-regulated miRNAs after UVA- and UVB-irradiation (miR-98, miR-323-3p, miR-330-3p, miR-376a, miR-494, miR-598) 3 miRNAs (miR-98, miR-330-3p and miR-376a) shared the common regulator element Hox-2.3-undefined-site-2 (gggggtgggggggag) in their promoter regions. No common regulatory elements were identified in the promoters for those miRNAs, which showed a response solely to UVA or UVB (up-regulation by UVA or UVB, down-regulation by UVA or UVB, in Table 1 and 2). This finding might be explained by the hypothesis that miRNAs, which are regulated by UVA or UVB alone, are involved in different specific stress response pathways (depending on the different UV radiation qualities) which need specific miRNAs with different regulatory elements to be regulated. On the other hand, those miRNAs, which are regulated both by UVA and UVB might be involved in critical steps of stress response pathways (e.g. defence of an increase in reactive oxygen species), which are activated by both radiation qualities, and, therefore, need miRNAs to be regulated with common regulatory elements.

*Thyroid hormone receptor T3R* is the only identified regulatory factor for GLYCA-nTRE (gcaggtgaggacttca) (shared by miR-191 and miR-376c, see above) [35]. Transcriptional regulation (e.g. of the cellular retinoic acid binding protein I) through the interaction of T3R with TRE is accompanied by chromatin remodelling [36]. Whereas the regulatory effect of T3R-TRE interaction on expression of many coding genes has been addressed [37], the function of TRE in the promoter of microRNAs has only been sporadically investigated. Recently it has been reported that T3R-binding to its TRE regulatory element in the promoter region of the miR-21 gene stimulates overexpression of the miRNA, thereby enhancing migration and invasion of hepatoma [38]. Thyroid hormone T3 also increases miR-34a expression (via TRE binding) and thus inhibits TGFβ1 induced renal tubular epithelial to mesenchymal transition [39]. Further, enhanced tumor metastasis in human hepatoma cells by thyroid hormone receptor has been caused by its repressive binding to TRE of miR-17 [40]. Using TLDA containing 600 rodent miRNAs Dong *et al.* identified altered expression for 40 miRNAs in the livers of hypothyroid mice compared to euthyroid controls [41]. All these indicate that the regulatory TRE element is used to stimulate/regulate other miRNAs (e.g. miR-21, one of the most described oncomiRs) involved in cancer associated processes.

### In silico analysis of UV-regulated miRNAs

In order to get an impression about possible genes and pathways which might be targeted by differentially expressed miRNAs after irradiation of human primary keratinocytes with UVA and UVB we did a gene ontology (GO) analysis of putative target genes (see Table S1). Genes in GO:0005515 (“protein binding”) are targeted with the highest frequency by miRNAs up-regulated after UVA or UVB irradiation (4 miRNAs). Two miRNA target genes belong to G0:0050789 (“regulation of biological process”). For miRNAs down-regulated after UVA or UVB again GO:0005515 (“protein binding”) is most frequently targeted by UV-modulated miRNAs (12 miRNA). GO:0043231 (“intracellular membrane bound organelle”) is the second frequent category (5 miRNAs).

**Figure 4:**
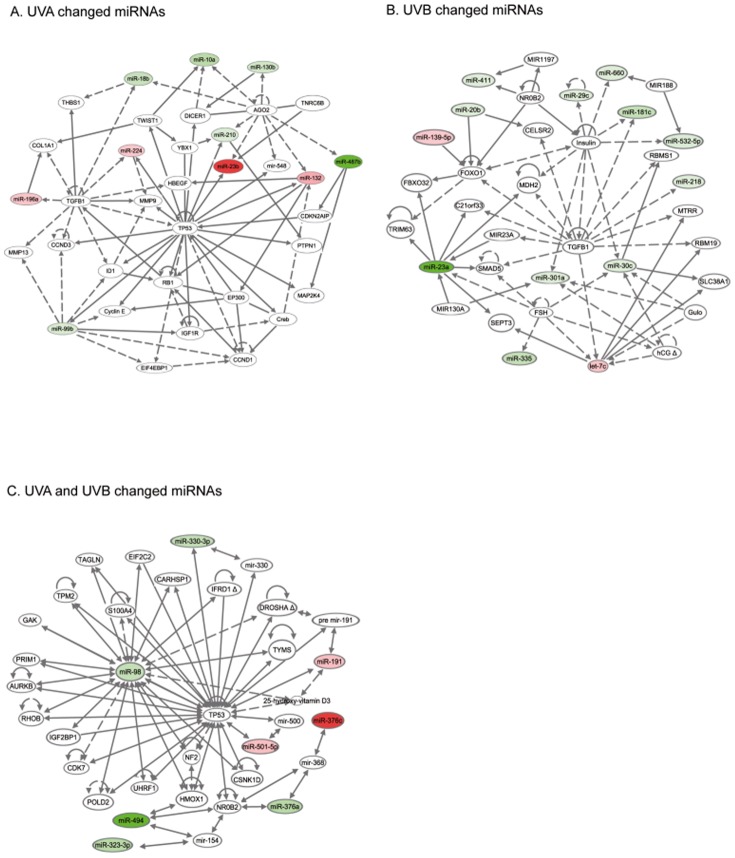
Schematic representation of a combination of the most significant networks after UV irradiation using Ingenuity Pathway Analysis of deregulated miRNAs. (A) The network “Cancer, Endocrine System Disorders, Gastrointestinal Disease” had a highly significant score of 24 after UVA irradiation. (B) After UVB irradiation the network “Reproductive System Disease, Cancer, Cardiovascular Disease” with a score of 35 is shown. (C) Network analysis of miRNAs similarly changed after UVA and UVB radiation identified “Connective Tissue Disorders, Inflammatory Disease, Inflammatory Response” as most significant network (score 14). A solid line represents a direct interaction and a dashed line indicates an indirect interaction. Colored molecules represent miRNAs found to be deregulated in our study (green: down-regulated; red: up-regulated).

To analyze miRNA-regulated pathways and networks involved in UV radiation response, the differentially expressed miRNAs after UVA and UVB were analyzed with Ingenuity pathway analysis (IPA) software. After UVA the most significant network affected is “Cancer, Endocrine System Disorders, Gastrointestinal Disease” with a highly significant score of 24. The network represents 10 focus molecules (miRNAs showing altered expression levels in our investigation), the tumor suppressor protein p53 (TP53) and TGF beta 1 are included as nodal molecules (Fig 4A). Cellular growth and proliferation and cellular development were the most affected biological pathways influenced by UVA-altered miRNAs.

Pathway analysis after UVB revealed two highly significantly networks “Reproductive System Disease, Cancer, Cardiovascular Disease” and “Cell Cycle, Cellular Growth and Proliferation, Cancer” (see Fig. 4B) with scores of 35 and 13. Here the most significant network includes 13 focus molecules. As nodal molecules TGF beta 1, insulin and FOXO1 were suggested. Cell growth and proliferation and cell death were the most influenced biological pathways. The nodal molecule TGF-beta 1 is already known to be regulated by UVB irradiation and it has functions in skin inflammation and carcinogenesis [42], [43].

The network analysis of miRNAs commonly changed after UVA and UVB irradiation revealed “Connective Tissue Disorders, Inflammatory Disease, Inflammatory Response” as the most significant network with a score of 14. The network includes eight of ten deregulated miRNAs triggered by UVA and UVB irradiation and displayed TP53 as nodal molecule (Fig. 4C). Cell cycle and cellular movement are identified as most impaired biological pathways.

Further, IPA network analysis showed that TP53 is connected to the subset of UVA as well as to the subset of UVA and UVB regulated miRNAs (Fig. 4A, C). This emphasizes the prominent function of TP53 for the response to both radiation qualities and suggests that the well established functions of the tumor suppressor protein TP53 in UV response including cell cycle control and apoptosis [44], [45] are mediated, at least in part, through miRNA-facilitated regulation. It is also well known that the tumor suppressor TP53 is often affected by UV signature mutations in skin cancer (especially in SCC) [46], [47].

To gain insights into the possible roles of most evidently changed miRNAs we performed a systematic analysis of the current literature in the context of skin cancer induction, development and UV-response. This analysis is summarized in Table 3. Because of its importance in skin development and skin cancers, miR-23b (up-regulated with a factor of 6.8 after UVA irradiation in our experiments) has been analyzed below in more detail.

**Table 3:** Proposed function of the most evidently changed miRNAs after UVA and UVB irradiation.

microRNA	proposed function	literature
**miR-376c (UVA +7.0)**	- increases proliferation, survival and chemoresistance- reduced 12 hours after UVB irradiation in mouse NIH3T3 cells	[22] [61]
**miR-494 (UVA** –**9.8)**	- may function as an oncogene in carcinogenesis by targeting several components related to cell cycle control and apoptosis (e.g. Pten, Kit)	[62], [63], [64], [65]
	- suppresses cell proliferation and induces senescence	
	- overexpression of miR-494 lead to down-regulation of Cdk6 and enhanced G1 arrest	
**miR-501-5p (UVB +6.8)**	- biomarker for melanoma diagnosis	[66]
**miR-361-5p (UVB +8.4)**	- involved in cutaneous squamous cell carcinoma (SCC)	[67]
**miR-23a (UVB** –**25.0)**	- slightly upregulated at 4h after the UVB irradiation of HaCaT cells, where it regulates UV-induced CPD removal, apoptosis and topoisomerase1\caspase7\STK4 expression	[68], [69], [70], [71], [72]
	- differentially expressed in melanocytes compared to melanoma cell lines and melanoma	
	- down regulation of miR-23a increases TNF-alpha- induced endothelial cell apoptosis	
	- anti-apoptotic and proliferation-promoting factor in liver cancer cells	
	- induced in human keratinocytes after UVB	
**miR-23b**	- targets deregulated mRNAs in BCC	[48] [73], [74], [75], [76]
**(UVA +6.8)**	- upregulated in human fibroblasts after UVC irradiation	
	- mediates the multiple steps of metastasis	
	- differentiation marker of human epidermal keratinocytes	
	- reduced in human melanoma samples	
**miR-191 (UVA +2.1) (UVB +1.7)**	- epigenetic upregulation of miR-191 promotes the transition of epithelial-to-mesenchymal in hepatocellular carcinoma	[77], [78]
	- triggers senescence in keratinocytes by Satb1 and Cdk6 down-regulation	
**miR-98 (UVA** –**3.1) (UVB** –**3.4)**	- involved in the regulation of cell proliferation and apoptosis by influencing the p53 pathway	[79]

### MicroRNA miR-23b

Using a new integrative software tool, miTRAIL, which allows comprehensive analyses of interactions of genes and miRNA based expression profiles, Laczny and coworkers have recently been able to show that human miR-23b targets the highest number of deregulated mRNAs and regulates the pathway “basal cell carcinoma, (BCC)” [48].

This analysis already indicates that miR-23b is an important miRNA involved in skin cancer development. As shown above, miR-23b is increased in expression with a factor of +6.8 after UVA-irradiation which is known to be an important risk factor for developing non-melanocytic skin cancers (NMSCs), like BCC [49], [50].

In accordance with our findings for UVA, miR-23b was also up-regulated after UVC irradiation of human fibroblasts [21] and after UVB irradiation of human keratinocytes (Zhou *et al*., 2012). We found an up-regulation of 1.6 after UVB irradiation, which is slightly below the cutoff (1.7) for regulated miRNAs in our study. Zhang *et al*. suggested that miR-23b suppresses multiple steps of metastasis including tumor growth, invasion and angiogenesis by the regulation of a cohort of prometastatic targets [51]. Most interestingly, miR-23b belongs to a group of 9 miRNAs, which are responsible for keratinocyte differentiation in human skin. Increased levels of miR-23b have been shown to represent a potent differentiation marker of human epidermal keratinocytes [52]. Furthermore, miR-23b was shown to be markedly reduced in human melanoma samples [53]. Therefore our findings might indicate that UVA irradiation triggers a (accelerated) differentiation program in human primary keratinocytes.

### Identification of direct targets of miR-23b in keratinocytes

To unveil the molecular mechanism of miR-23b, we aimed to identify direct target genes. We used three algorithms that predict the targets of the miRNA: PicTar, TargetScan and miRDB and a literature research based on PubMed references. Out of the list of putative targets RRAS2, TGFβR2 (transforming growth factor β receptor) and VHL (von Hippel Lindau) have a possible involvement in stress response or differentiation pathways [51], [54].

**Figure 5:**
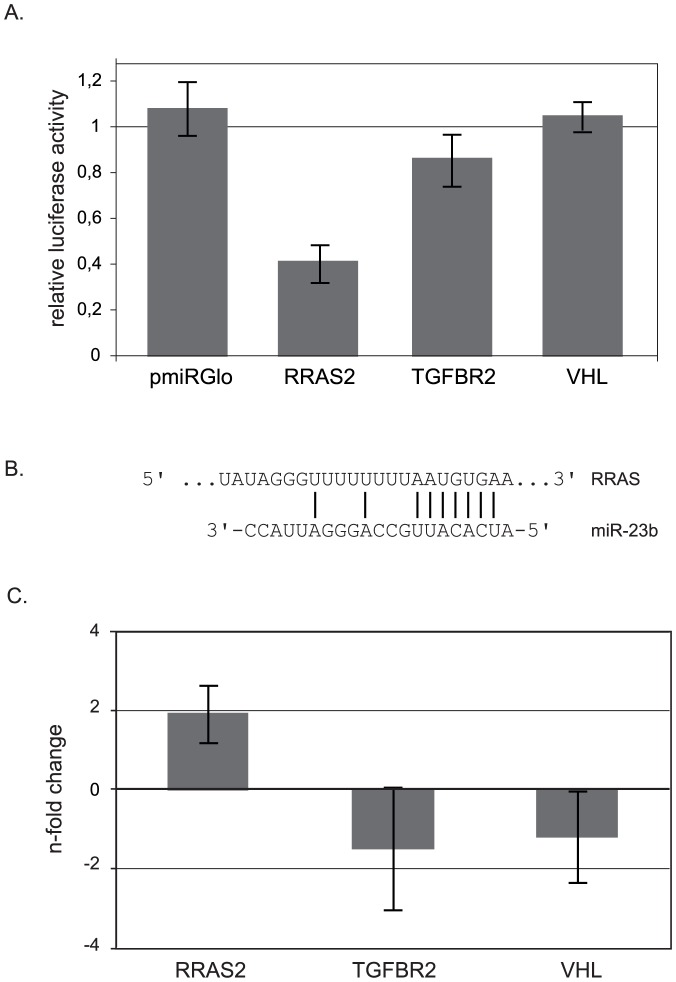
Identification of direct targets of miR-23b using luciferase assays. (A) Relative luciferase activities after co-transfection of luciferase constructs and control miRNA or pre-miR-23b in HaCaT cells. The firefly luciferase values were normalized for transfection with renilla luciferase activity. Relative luciferase activities represent the ratio between normalized luciferase activities of pre-miR-23b and control miRNA transfected cells. The mean ± s.e.m. of three independent experiments is shown. (B) Complementarity of miR-23b sequence to the RRAS2 gene sequence. Vertical lines indicate identity between miRNA sequence and corresponding gene sequence. (C) Transcriptional change of the putative targets (RRAS2, TGFBR2 and VHL) of miR-23b in human primary keratinocytes after UVA treatment (600 kJ/m^2^, 6h post irradiation) was analyzed via qPCR. Geometric mean of the expression of the house keeping genes: ACTB (beta actin), HPRT1 (hypoxanthine phosphoribosyltransferase 1) and TBP (TATA box binding protein) was used for normalization. Fold-change of the transcription upon UVA was obtained by setting the control as one-fold. Two-fold threshold was applied as criterion of altered transcriptional response. Error bars indicate standard deviations. N ≥ 3.

To access the direct interaction of miR-23b and RRAS2, TGFβR2 and VHL, we cloned the 3′-untranslated region (UTR) of these three putative miR-23b targets into a dual-luciferase reporter plasmid and performed a quantitative examination of the luciferase activities in precursor-miR-23b (pre-miR-23b) or control-miR transfected HaCaT cells. The empty reporter plasmid (pmirGlo) was used as control. For TGFRB1 and VHL reporter constructs only a weak or no reduction in luciferase activities was detected in pre-miR-23b transfected cells compared to control-miR transfected cells, indicating a lack of interaction between these targets and miR-23b. A significantly decreased luciferase activity was observed for the RRAS2 reporter construct in miR-23b precursor-miRNA transfected cells. This result confirmed the direct repression of the RRAS2 reporter by miR-23b, establishing it as a direct miR-23b target. A high complementarity between the 3’-UTR of RRAS2 and the seed sequence of miR-23b could be detected (Fig. 5A, B). Steady state mRNA levels of RRAS2 after UVA-irradiation, however, did not change significantly (Fig. 5C) suggesting that miR-23b influences the translation efficiency of RRAS2 mRNA rather than the mRNA stability. RRAS2 has been shown to mediate transformation and cell survival via the activation of Phosphatidylinositol-3-kinase and Nuclear factor κB [55]. Interestingly, expression of RRAS2 is highly correlated with the aggressiveness of malignant skin cancers and a UV-mediated regulation is suggested in human tumorigenic prostate cells [56], [57].

## Summary

In this study we show for the first time that there is a common miRNA response to UV accompanied by distinct wavelength-specific miRNA responses to UVA and UVB radiation. Whether the wavelength-specific pathways depend on e.g. different DNA damage profiles and/or different amounts and qualities of reactive intermediates like reactive oxygen species (ROS) after UVA or UVB irradiation has to be elucidated in future research.

Our results give the first hints that transcription of some miRNAs responsive to both UVA and UVB irradiation might be regulated via common regulatory elements. Further, analysis of the changed miRNAs and putative target genes indicate cell growth and proliferation as an important pathway, which is epigenetically regulated in cellular UV response. The related networks suggest miRNA regulation for the well-known p53-mediated DNA damage response pathway as well as for less characterized TGF-beta-mediated pathways in UV irradiation response.

UVA especially induced the expression of miR-23b, known to be a pleiotropic modulator of cancer metastasis and a marker of human keratinocyte differentiation, which plays a prominent role in epidermal stem cell fate and skin carcinogenesis [51]. Our results further show that the Ras related protein 2 gene (RRAS2) is a direct target.

Having in mind that UVA- and UVB-radiation have been acknowledged as “known carcinogens to humans” [17], our findings could contribute to further understanding of UVA and UVB-triggered, miRNA-mediated, epigenetic modulation of gene expression and its implications for photocarcinogenesis in human skin.

## Materials and Methods

### UV irradiation

Human primary keratinocytes from two female skin samples grown to confluence in KGM medium were subjected to UV irradiation. Human primary keratinocytes used in this study were obtained after the donors had given their written informed consent, which allows the use of skin material (biopsies) for scientific research in the field of photocarcinogenesis. An approval of the research has been obtained from the ethics committee of Elbekiliniken Stade/Buxtehude. For treatment with UVA (600 kJ/m^2^, 315–400 nm, peak emission λ_max_  =  365 nm) a UVA source (Philips HB404, equipped with an infrared filter and a Hoenle UVB blocking filter) was operated at a dose-rate of 183 W/m^2^. For UVB irradiation (300 J/m^2^, 280–315 nm, peak emission λ_max_  =  312 nm, dose rate 3.9 W/m^2^) we used an array of TL-12 lamps (Philips) in combination with a UVC blocking filter (Kodacell). UVA and UVB doses (600 kJ/m^2^ and 300 J/m^2^) produced a comparable number of CPDs, as detected by flow cytometry using monoclonal antibodies against CPD (see. Fig 1). Prior to irradiation the medium was removed and cells were washed once with PBS. During the UVA irradiation cells were covered with PBS, whereas no PBS was added onto cells for UVB irradiation. For both UVA and UVB irradiations cells were kept cold on the ice. Sham irradiated controls were kept under the same experimental conditions as their irradiated counterparts. After the irradiation, cells were grown for 6h before RNA isolation.

### miRNA isolation

miRNA was isolated with the mirVana™ miRNA Isolation Kit (Ambion, Austin, TX), according to the manufacturer’s protocol. The quality and the concentration of the RNA samples were determined with Infinite 200 NanoQuant (Tecan, Switzerland). The RNA quality for the array analysis ranges between 1.9 and 2.05 using an OD 260/280 ratio. RNA quality was also checked by RNA integrity analysis in the 2100 Bioanalyzer (Agilent Technologies, USA) where RNA integrity numbers (RIN) between 9.8–9.5 indicative of high integrity were achieved.

### Reversed transcription and microarray analysis of miRNA profiles

450 ng of total RNA was reverse transcribed using a Human Multiplex RT Primer Set and the TaqMan® MicroRNA Reverse Transcription Kit (Applied Biosystems, Forster City, CA). Samples (reaction volume 7.5 µl) were incubated for 40 cycles in a thermocycler for 2 min at 16°C, 1 min at 42°C, 1 min at 85°C, followed by a 5 min step at 85°C and then held at 4°C. Global profiling of miRNA expression was performed using the TaqMan Low Density Array (TLDA) (Applied Biosystems, Forster City, CA) assaying 378 human miRNA sequences, plus three small nucleolar RNAs (snoRNAs): *RNU6B, RNU48* and *RNU44* as endogenous controls. Large-scale polymerase chain reaction (PCR) of RNA from sham, UVA and UVB treated cells were performed using a standard TaqMan PCR kit protocol on a 7900HT Fast Real-Time PCR System (Applied Biosystems) containing 450 µl TaqMan Universal PCR Master-Mix, 6 µl RT-PCR products and 444 µl nuclease-free water was applied to the TLDA and PCR was performed at 95°C for 10 min, followed by 40 cycles of 95°C for 15 seconds and 60°C for 1 min. Data were acquired and analyzed using the Sequence Detection System software (v. 2.3) (Applied Biosystems). A cut-off of 32 was applied to discard the late ct values. UVA/UVB-induced miRNA expression was calculated from the subset of miRNAs detected in sham and in irradiated samples by the comparative 2^−ΔΔCt^ method [58] with RNU6B, RNU44 and RNU48 as endogenous controls and normalization to the non-irradiated control. The validation of deregulated miRNAs was done by real-time PCR with miRNA specific primer setups according to the manufactures instructions (Applied Biosystems). Alterations in miRNA expression versus sham irradiated cells was considered significant if the p<0.05 for the three replicates and the expression changed by more than 1.5-fold compared to controls. microRNA expression data of TLDA are available in Table S3.

### Cell culture

The human keratinocyte cell line HaCaT was maintained in RPMI medium 1640 (PAA Laboratories) supplemented with 10% fetal calf serum and 2 mM glutamine. The cell line was grown at 37°C in a humified atmosphere of 5% CO_2_.

### Luciferase reporter assay to identify mRNAs directly targeted by miR-23b

cDNA sequences of candidate gene were obtained by PCR amplification of reverse transcribed mRNA derived from primary human keratinocytes using the following primer sets: VHL-For, and VHL-Rev; RRAS2-For and RRAS2-Rev; TGFBR2-For and TGFBR2-Rev (sequences see Table S2). The PCR fragments were directly cloned into the pmirGLO Dual-Luciferase miRNA Target Expression Vector (Promega, USA) using the PmeI and SbfI restriction sites. The vector uses dual-luciferase technology, with Firefly luciferase (luc2) being the reporter used to quantify miRNA regulation of translation and Renilla luciferase (hRluc-neo) being the non-regulated internal control. The identity and integrity of all constructs were confirmed by DNA sequencing.

Transfection of reporter constructs into the human keratinocyte cell line HaCaT was performed using Lipofectamine 2000 (Invitrogen, USA) in duplicate 96-well plates. Five nmol of either pre-miR-23b (precursor-miR-23b, processed to mature miR-23b by endogenous Dicer after transfection) or the unspecific control oligonucleotides (control-miR) were transfected along with the 0.2 µg pmirGlo Dual-Luciferase construct harboring cDNA of putative miR-23b regulated targets. Forty eight hours post transfection, cells were lysed with passive lysis buffer and the activities of Firefly luciferase and Renilla luciferase were measured using the dual Luciferase Assay System (Promega, USA). The ratio of Firefly luciferase and Renilla luciferase was expressed as normalized luciferase activity to compensate differences in transfection efficiencies. The relative luciferase activity was determined as the ratio between normalized luciferase activities of cells transfected with pre-miR-23b and control miRNA.

### Transcriptional analysis of putative targets of miR-23b in human keratinocytes after UVA treatment

Total RNA was isolated from un-irradiated and UVA-irradiated human keratinocytes (600 kJ/m^2^, 6h post irradiation, see above) and cDNA synthesis (using the “Enhanced Avian First Strand Synthesis Kit”, Sigma-Aldrich) was performed according to the protocols of the manufacturer. cDNA was quantified with qPCR using Realplex-Mastercycler (Eppendorf). For PCR reactions a SYBR Green containing Mix (SensiMix, Bioline) was applied. Geometric mean of the expression of the house keeping genes: ACTB (beta actin), HPRT1 (hypoxanthine phosphoribosyltransferase 1) and TBP (TATA box binding protein) was used for normalization. Fold-change of the transcription upon UVA was obtained by setting the control as one-fold. Two-fold threshold was applied as criterion of altered transcriptional response. Primers used are given in Table S2.

### Prediction of potential regulatory elements using the NSITE program

Potential regulatory elements in the promoter region (1500 bp) of the miRNAs commonly regulated by UVA and UVB were identified using the NSITE program (Softberry,

http://linux1.softberry.com/berry.phtml?topic=nsitem&group=programs&subgroup=promoter, using the Ghosh database). As input the up-regulated miRNA-set consists of miR-191, miR-376c and miR-501-5p. The down-regulated miRNA-set consists of miR-98, miR-323-3p, miR-330-3p, miR-376a, miR-494 and miR-598**.**

### Bioinformatic analysis

For gene ontology analysis putative miRNA targets were identified using miRBase (www.mirbase.org), PicTar (www.pictar.mdc-berlin.de) and TargetScan (www.target.sca.org). Lists of candidate genes were then uploaded into a GO analysis program (www.gostat.wehi.edu.au). In this way, for each miRNA the highest ranking GO categories were determined. Target genes for each GO category (ranging in number from a few to several hundred) were listed and the highest ranking gene in that list was chosen as the target gene that was described in more detail**.** Only those miRNAs with expression change >2 fold have been used for the GO analysis (Table S1).

To obtain information about biological mechanism, pathways and network relationships of differentially regulated miRNAs Ingenuity Pathway Analysis (IPA) analysis was used (Ingenuity Systems, http://www.INGENUITY.com). For this purpose all differentially regulated miRNAs and fold change were imported into IPA [59]. The statistical probability of a pathway being randomly identified was determined by calculating the p-value using Fisheŕs exact test. Scores represent the logarithm of the probability that the network would be found by chance; scores ≥2 are considered to be significant.

## Supporting Information

Table S1 Gene Ontology (GO) analysis of miRNA which are differentially expressed (> 2 fold) in human primary keratinocytes 6 h after UV-A irradiation.
(DOCX)

Table S2 Primers used in this study.
(XLSX)

Table S3 microRNA expression data of TLDA.
(XLSX)
