# Supplementary material for: UVA and UVB Irradiation Differentially Regulate microRNA Expression in Human Primary Keratinocytes
Source: PLoS One. 2013 Dec 31;8(12):e83392. doi: 10.1371/journal.pone.0083392 (PMC3877020; doi:10.1371/journal.pone.0083392)
Supplement: Table S1 — Gene Ontology (GO) analysis of miRNA which are differentially expressed (> 2 fold) in human primary keratinocytes 6 h after UV-A irradiation. (DOCX) [file pone.0083392.s001.docx]

Supplementary Table 1:

Gene Ontology (GO) analysis of miRNA which are differentially expressed (> 2 fold) in human primary keratinocytes 6 h after UV-A irradiation. Underlined miRNAs are changed after UV-A and UV-B irradiation.

| **UV-A up** | **GO-specification** | **First gene in GO** | **name** | **function** |
| --- | --- | --- | --- | --- |
| miR-23b | **GO:0005515**  Protein binding  P=5.6e-24 | Mapre3 | Microtubule-associated protein, RP/EB family member | Involved in microtubule polymerization and spindle function by stabilizing microtubules and anchoring them at the centromere. May play a role in cell migration. |
| miR-132 | **GO:0048523**  Negative regulation of cellular process  P=1.3e-15 | Ppm1g | Protein phosphatase 1G (formerly 2C) | Member of the PP2C family of Ser/Thr protein phosphatases. PP2C family members are negative regulators of cell stress response pathways. PP2CG has been suggested as a regulator of p21(Cip1/WAF1) protein stability via the Akt signaling pathway. |
| miR-191 | **GO:000725**  Multicellular organismal development  P=6.1e-4 | Atp2b2 | ATP Ca^++^ plasmamembrane pump | Plays a role in intracellular calcium homeostasis. An association between ATP2B2 gene variants and autism in male patients has been described. |
| miR-340 | **GO:0000163**  Protein phosphatase type 1 activity  P=2.97e-2 | Ppp1cb | Protein phosphatase1 catalytic subunit | Serine/threonine specific phosphatase involved in cell division, glycogen metabolism, muscle contractility, protein synthesis. Overexpressed in malignant melanoma. |
| miR-376c | **GO:0050789**  Regulation of biological process  P=6.4e-15 | En2 | Engrailed homeobox 2 | Encodes homeo-domain-containing proteins, implicated in the control of pattern formation during development of the central nervous system. |
| miR-501-5p | **GO:0005515**  Protein binding  P=1.4e-12 | Phf17 | PHD (plant homeo-domain) finger protein 17  Also know as gene for apoptosis and differentiation in epithelia (JADE1) | Inhibits Wnt-signaling by ubiquitination of β-catenin. Component of the HBO1 complex which has a histone H4-specific acetyltransferase activity. |
| miR-452 | **GO:0005515**  Protein binding  P=3.0e-18 | Ank2 | Ankyrin 2, neuronal | Ankyrins play an essential role in activities such as cell mobility, activation, proliferation and contact. |
| **UV-A down** | **GO-specification** | **First gene in GO** | **name** | **function** |
| miR-10a | **GO:0006355**  Regulation of transcription, DNA dependent  P=5.4e-16 | Baz2b | Bromo-domain adjacent zinc finger domain 2b | May play a role in transcriptional regulation of ISWI. |
| miR-18b | **GO:0005515**  Protein binding  P=1.3e-13 | Pard6b | Par-6 partitioning 6 homolog beta (*s. elegans*) | Involved in asymmetrical cell division. |
| miR-98  (see let-7c) | **GO:0005515**  Protein binding  P=2.7e-20 | Rasgrp1 | RAS guanyl releasing protein 1 | Functions as a diacylglycerol (DAG)-regulated nucleotide exchange factor specifically activating Ras through the exchange of bound GDP for GTP. |
| miR-99b | **GO:0044424**  Intracellular part  P=1.75e-2 | Mycbp2 | Myc binding protein 2 | E3 ubiquitin protein ligase. Mediates the ubiquitination and subsequent proteosomal degradation of target proteins. May function as a facilitator or regulator of transcriptional activation of myc. |
| miR130b | **GO:0005515**  Protein binding  P=1.4e-39 | Btbd10 | BTB (POZ) domain containing 10 | Mediator of interactions among proteins involved in transcriptional regulation and chromatin structures. Down regulated in glioma. |
| miR-323-3p | **GO:0043231**  Intracellular membrane-bound organelle  P=6.3e-13 | Gatad2b | GATA zinc finger domain containing 2B | Transcriptional repressor activity. Binds methyl-CpG-binding domain proteins MDB2 and 3. |
| miR-376a | **GO:0043231**  Intracellular membrane-bound organelle  P=2.2e-3 | Rpl5 | Ribosomal protein L5 | Required for rRNA maturation and formation of 60 S ribosomal subunits. Binds 5SRNA. |
| miR-330-3p | **GO:0005515**  Protein binding  P=4.9e-28 | Pcdh11x | Protocadherin 11 x-ligated | Potential calcium-dependent adhesion protein. Plays a fundamental role in cell-cell recognition. Essential for the segmental development and function of nervous system. |
| miR-487b | **GO:0043231**  Intracellular membrane-bound organelle  P=9.3e-3 | Sap30l | 2in3A associated protein p30-lik3 (histone deacetylase complex subunit | Involved in the functional recruitment of the class 1 Sin3-histone deacetylase complex (HDAC) to the nucleolus. |
| miR-494 | **GO:0050794**  Regulation of cellular process  P=2.3e-26 | Cul1 | Cullin1 | Core component of multiple cullin-RING-based SCF E3 ubiquitin-protein ligase complexes. Involved in cell cycle progression, signal transduction and transcription |
| miR-598 | **GO:0000318**  Protein-methionine R-oxidase reductase activity  P=1.7e-3 | Msrb3 | Methionine sulfoxide reductase B3 | Expression in epidermal keratinocytes and melanocytes. Differentially expressed in human skin. May be involved in human skin aging and carcinogenesis. |

Gene Ontology (GO) analysis of miRNA which are differentially expressed (> 2 fold) in human primary keratinocytes 6 h after UV-B irradiation. Underlined miRNAs are changed after UV-A and UV-B irradiation.

| **UV-B up** | **GO-specification** | **First gene in GO** | **name** | **function** |
| --- | --- | --- | --- | --- |
| Let-7c | **GO:0005515**  Protein binding  P=2.7e-20 | Rasgrp1 | RAS guanyl releasing protein 1 | Functions as a diacylglycerol (DAG)-regulated nucleotide exchange factor specifically activating Ras through the exchange of bound GDP for GTP. |
| miR-139-5p | **GO:0048856**  Anatomical structure development  P=1.9e-21 | Shc1 | SHC (Scr homology 2 domain containing) transforming protein 1 | Involved in regulation of apoptosis and drug resistance in mammalian cells. |
| miR-361 | **GO:0043231**  Intracellular membrane-bound organelle  P=7.6e-14 | Tfap2b | Transcription factor AP-2 beta (activating enhancer binding protein beta 2) | Member of the AP-2 family of transcription factors stimulating cell proliferation and suppressing terminal differentiation of specific cell types during embryonic development. |
| miR-362-5p | **GO:0032502**  Developmental process  P=4.8e-20 | Dpysl3 | Dihydro-pyrimidase-like 3 | Necessary for signaling by class 3 semaphorins and subsequent remodeling of cytoskeleton. Plays a role in axon guidance, neuronal growth core collapse and cell migration. |
| miR-376c | **GO:0050789**  Regulation of biological process  P=6.4e-15 | En2 | Engrailed homeobox 2 | Encodes homeo-domain-containing proteins, which have been implicated in the control of pattern formation during development of the central nervous system. |
| miR-501-5p | **GO:0044424**  Intracellular part  P=5.0e-10 | Slc25a43 | Solute carrier family 24, member 43 | Encodes a member of mitochondrial carrier family of proteins. |
| **UV-B down** | **GO-specification** | **First gene in GO** | **name** | **function** |
| miR-20b | **GO:0044424**  Intracellular part  P=4.6e-54 | Lass6 | LAG 1 longevity assurance homolog 6 | May be involved in sphingolipid synthesis or its regulation. |
| miR-23a | **GO:0005515**  Protein binding  p=2.93e-31 | Kpna1 | Karyopherin α1 (Importin α1) | Functions in nuclear protein transport. Interacts with recombination activation protein RAG1 and RAG2. Kpna1 has been shown to play a role in V(D)J recombination. |
| miR-29c  (see also miR-501-5p) | **GO:0032502**  Developmental process  P=1.8e-28 | Phf17 | PHD finger protein 17 | Component of the HBO1 complex which has a histone h4-specific acetyltransferase activity. |
| miR-96 | **GO:0005515**  Protein binding  P=3.0e-46 | Snx13 | Sorting nexin 13 | RGS domain and PHOX domain-containing protein. Involved in intracellular trafficking. Overexpression delayes lysosomal degradation of EGFR. |
| miR-98 | **GO:0005515**  Protein binding  P=2.7e-20 | Rasgrp1 | RAS guanyl releasing protein 1 (Calcium and DAG regulated | Function as a diacylglycerol (DAG)-regulated nucleotide exchange factor specifically activating RAS through target exchange of GDP for GTP. |
| miR-181c | **GO:0005515**  Protein binding  P=1.0e-41 | sacs | Spastic ataxia of Charlevoix-Saguenay (Sacsin) | May function in chaperone mediated protein folding. Encodes the Sacsin protein. |
| miR-323-3p | **GO:0043231**  Intracellular membrane-bound organelle  P=6.3e-13 | Gatad2b | GATA zinc finger domain containing 2B | Transcriptional repressor activity. Binds methyl-CpG-binding domain proteins MDB2 and 3. |
| miR-330-3p | **GO:0005515**  Protein binding  P=4.9e-28 | Pcdh11x | Protocadherin 11 x-ligated | Potential calcium-dependent adhesion protein. Plays a fundamental role in cell-cell recognition. Essential for the segmental development and function of nervous system. |
| miR-335 | **GO:0005515**  Protein binding  P=1.42e-11 | Wwp1 | WW domain containing E3 ubiquitin protein ligase1 | Important role in regulation of a variety of cellular functions such as protein degradation, transcriptional splicing. Ubiquitinates e.g. TP63. |
| miR-376-a | **GO:0043231**  Intracellular membrane-bound organelle  P=2.2e-3 | Rpl5 | Ribosomal protein L5 | Required for rRNA maturation and formation of 60 S ribosomal subunits. Binds 5SRNA. |
| miR-411 | **GO:0005515**  Protein binding  P=1.1e-8 | Pou4f1 | POU class 4 homeobox | Class IV POU domain-containing transcription factor highly expressed in developing sensory nervous system. |
| miR-503 | **GO:0005515**  Protein binding  P=2.7e-24 | Pcdha6 | Protocadherin alpha 6 | Potential Ca-dependent cell-adhesion protein. May be involved in the establishment and maintenance of specific neuronal connections in the brain. |
| miR-532-5p | **GO:007275**  Multicellular organismal development  P=7.5e-15 | hunk | Hormonally up-regulated Neu-associated kinase | Hunk is required for HER2/neu-induced mammary tumorigenesis. |
| miR-660 | **GO:0005515**  Protein binding  P=1.6e-6 | cnot6l | CCR4-NOT transcription complex, subunit 6 like | Plays a role in deadenylation of mRNAs in the cytoplasm. Involved in deadenylation-dependet degradation of CDKN1B mRNA. |

Characterization of miRNA was done by *miRNAbase* ([www.miRBase.org](http://www.miRBase.org)) and *GOsta*t ([www.gostat.wehi.edu.au](http://www.gostat.wehi.edu.au)). Search for miRNA target genes has been performed by using the *PicTar webinterface* (<http://pictar.mdc-berlin.de>) and then used for gene ontology (GO) analysis. p-values are taken from *mirbase-, gostat- and pictar*-statistics.
